# Addendum: Sensing the allosteric force

**DOI:** 10.1038/s41467-023-37893-z

**Published:** 2023-05-12

**Authors:** Brankica Jankovic, Olga Bozovic, Peter Hamm

**Affiliations:** Department of Chemistry, University of Zurich, Zurich, Switzerland

Addendum to: *Nature Communications* 10.1038/s41467-020-19689-7, published online 17 November 2020

In a recent paper^1^, we studied the allosteric mechanisms of a photoswitchable PDZ3 domain. The design of the system was motivated by previous works^2^, in which it has been shown that removing the terminal *α*3-helix of the PDZ3 domain, or disturbing its structure by phosphorylation, significantly affects the binding affinity of small peptides to the binding groove of the protein. We instead covalently linked a photo-isomerizable azobenzene moiety to the terminal *α*3-helix, designed in a way that the *α*-helical structure is stabilized in the *cis*-state of the photoswitch, and destabilized in the *trans*-state. This allowed us to reversibly switch between two states of the protein with the help of light. We showed by CD spectroscopy that the helical content of the protein indeed changed in an anticipated way (Fig. 2a in ref. ^1^), we measured the binding affinities of a small pentapeptide in the *cis* and the *trans*-state of the azobenzene moiety with the help of fluorescence quenching experiments (Fig. 2b in ref. ^1^), as well as the thermally driven *cis*-to-*trans* isomerization rates with and without a ligand bound to protein (Fig. 2c in ref. ^1^). From Van-t’Hoff and Arrhenius plots (Fig. 3 in ref. ^1^), the energetics of the allosteric cycle has been deduced and the ligand-induced force the protein exerts on the azobenzene moiety has been determined. The work contains two errors, one in the measurement of the binding affinities by fluorescence quenching, and a second conceptual one in determining the energetics. In this Addendum, we wish to address both errors.

## Binding affinity measured by fluorescence quenching

In ref. ^1^, we measured the binding affinity by fluorescence quenching. The fluorescence originated from tryptophan in the pentapeptide ligand, whose yield increases when the peptide binds to the protein, presumably due to a more rigid structure. We kept the concentration of the peptide constant (15 μM), varied that of the protein between 0 and 50 μM, and fitted the resulting fluorescence signal to a two-state binding equilibrium. The excitation and detection wavelengths were set to 250 and 325 nm, respectively, both isosbestic points where the absorption of the azobenzene moiety is the same in the *cis* and the *trans*-states.

We, however, completely underestimated the effect of (re-)absorption. That is, as we increased the protein concentration in these experiments, its absorption due to the attached azobenzene moiety increased as well. This affects both the excitation of the tryptophan by absorbing excitation light at 250 nm, as well as the detection of its emission at 325 nm by reabsorption. The red data in Fig. 1 (which have been remeasured with different concentrations and different wavelengths as in ref. ^1^, see figure caption for details) illustrate the problem. In the *cis*-state, the fluorescence signal initially rises due to the reduced quenching upon binding, but then decreases again due to absorption effects. In the *trans*-state, the initial rise, which is smaller due to the smaller binding affinity, is overcompensated by the reabsorption effect already at low concentrations. In ref. ^1^, we stopped at a protein concentration of 50 μM, and thus wrongly fitted the maximum in the fluorescence signal at this point as the anticipated asymptotic value in a binding equilibrium. In that way, we significantly overestimated the binding affinities, as well as the differences between *cis* and *trans*-states. Furthermore, since all involved processes are temperature dependent in a competing manner, the resulting temperature trends were wrong.

**Fig. 1:**
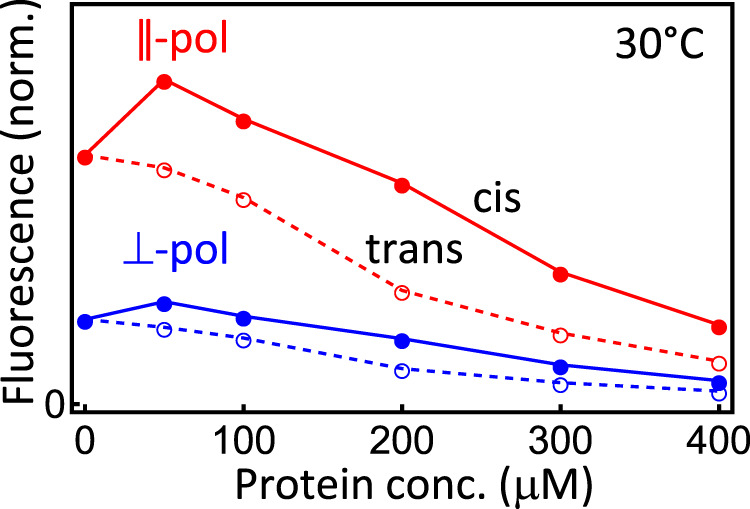
Fluorescence signal measured as a function of initial protein concentration. Shown are parallel (red) and perpendicular (blue) polarizations of excitation light and fluorescence detection, respectively, exemplified here for the *cis*-state (filled circles, solid lines) and *trans*-state (open circles, dashed lines), respectively, at 30 °C. The peptide concentration has been 100 μM, and the excitation and detection wavelengths have been set to 280 and 360 nm, respectively, in order to maximize the fluorescence signal (it is actually not necessary to work at isosbestic points).

We did not manage to reduce the sample thickness and/or sample concentration to the extent that the described absorption effects would be negligible; the amount of the resulting fluorescence light then was just too low to measure anything reasonable. Instead, we measured the fluorescence depolarization to determine the binding affinity^3^. The anisotropy is defined as (*I*_∥_ − *I*_⊥_)/(*I*_∥_ + 2*I*_⊥_)), where *I*_∥_ and *I*_⊥_ are the detected fluorescence signals for parallel and perpendicular polarization directions of excitation light vs emission detection, respectively (Fig. 1, red for parallel and blue for perpendicular polarization). The effects of absorption and reabsorption are canceled out in that way, since both happen in the bulk solution and thus do not have any polarization dependence. Since the unbound peptide is a relatively small molecule, it orientationally diffuses on the timescale of tryptophan fluorescence, and the detected anisotropy is smaller than the upper limit of 0.4. On the other hand, once it is bound to the protein, orientational diffusion does, in essence, no longer occur on this timescale due to the much larger size of the protein. Figure 2a shows the resulting anisotropies as a function of initial protein concentration and temperatures. A significant difference in binding between *cis* and *trans*-state can be observed, despite the fact that these data are more noisy than the raw fluorescence data of Fig. 1 (or those from Fig. 2b of ref. ^1^). They are more noisy since two data sets are put into relation with each other, and since the change in anisotropy between free and protein-bound peptide is very small. For the best possible comparability, *trans* and *cis*-states have therefore been measured directly after each other, for exactly the same sample and without touching anything except for switching on a 370 nm LED to promote the sample from the *trans* into its *cis*-state.

**Fig. 2:**
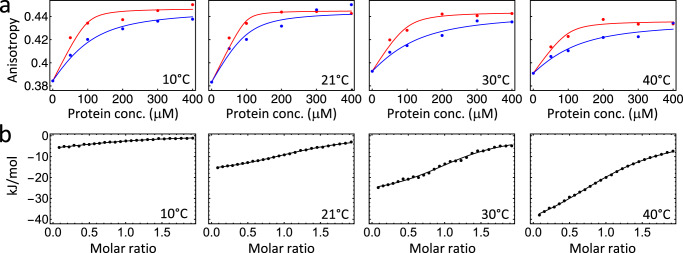
Fluorescence depolarisation and ITC measurements. **a** Fluorescence anisotropy as a function of initial protein concentration and temperature with the photoswitch in the *cis*-state (red) or the *trans*-state (blue). peptide concentration has been 100 μM in all experiments. The anisotropy of the bound state is a bit larger than the theoretical upper limit of 0.4 due to slightly different detection sensitivities of the used fluorometer for both polarization directions. **b** ITC measurements of the *trans*-state. In either case, the data have been fit to a two-state binding equilibrium (solid lines).

Fitting a two-state binding equilibrium to the data, the binding affinities can be extracted, which are listed in the upper half of Table 1. In optimizing the measurement parameters, one critical issue has been to be able to measure at a protein concentration high enough, despite reabsorption, so that the plateau of full binding is reached; otherwise, the fit would not have been stable. The concentrations that were needed to obtain a sufficient amount of fluorescence are significantly larger than the dissociation constant for the *cis*-state, hence its error is relatively large. Nonetheless, it is clear that the binding affinity of the *cis*-state is significantly larger at all temperatures by about a factor of 8 ± 2, which amounts to a binding free energy that is larger by $\Delta \Delta G=RT\ln {K}_{d,cis}/{K}_{d,trans} \, \approx \, 5.2\pm 0.5$ kJ/mol.

**Table 1:** Binding affinities in the *cis* and the *trans*-state, as determined from fluorescence depolarization (upper half), as well as binding enthalpy and binding free energy in the *trans*-state, as determined from ITC (lower half)

	10 °C	21 °C	30 °C	40 °C
*K*_*d*,*cis*_ (μM)	5 ± 7	2 ± 3	9 ± 7	8 ± 7
*K*_*d*,*trans*_ (μM)	42 ± 5	17 ± 8	66 ± 10	61 ± 13
Δ*H*_*trans*_ (kJ/mol)	−9.3 ± 1.0	−18.3 ± 0.3	−28.5 ± 0.7	−49.7 ± 0.7
Δ*G*_*trans*_ (kJ/mol)	−19.3 ± 0.4	−22.0 ± 0.4	−23.4 ± 0.2	−22.5 ± 0.4

## Determination of the energetics

Besides the error in the data accumulation described above, there has also been a conceptual mistake in the data analysis. That is, we used the temperature-dependent binding affinities to disentangle the binding free energy, Δ*G*, into its enthalpic and entropic contributions via Δ*G* = Δ*H* − *T*Δ*S*. To that end, we implicitly assumed that Δ*H* is temperature independent and that the temperature dependence of Δ*G* is dominated by the explicit *T*-factor in the equation above. That assumption is inherently wrong for a protein-ligand association, as discussed in a number of publications.^4–6^

In fact, in order to verify the new binding affinities measured by fluorescence depolarization (Fig. 2a), we also used ITC as an alternative method, see Fig. 2b. In contrast to fluorescence depolarization, which reveals only the binding free energy, ITC measures Δ*H* (i.e., the explicit measurand of ITC) and Δ*G* (via the slope of the data in Fig. 2b) separately for each individual temperature, see Table 1, lower half. ITC is the only method that can measure Δ*H* of binding directly and independently from Δ*G*, and hence does not have to rest on any assumption. We find that the binding enthalpy, Δ*H*, and the entropic contribution, − *T*Δ*S*, are strongly temperature dependent, albeit in a way that the temperature dependence of the binding free energy, Δ*G*, largely cancels out in the considered temperature range (Fig. 3). This effect is known as “entropy-enthalpy compensation”.^4–6^ To a certain extent accidentally, the linear term of both contributions cancel out, but higher order terms remain, hence the resulting free energy Δ*G* is curved in the considered temperature range (see e.g., Fig. 3 in ref. ^4^).

**Fig. 3:**
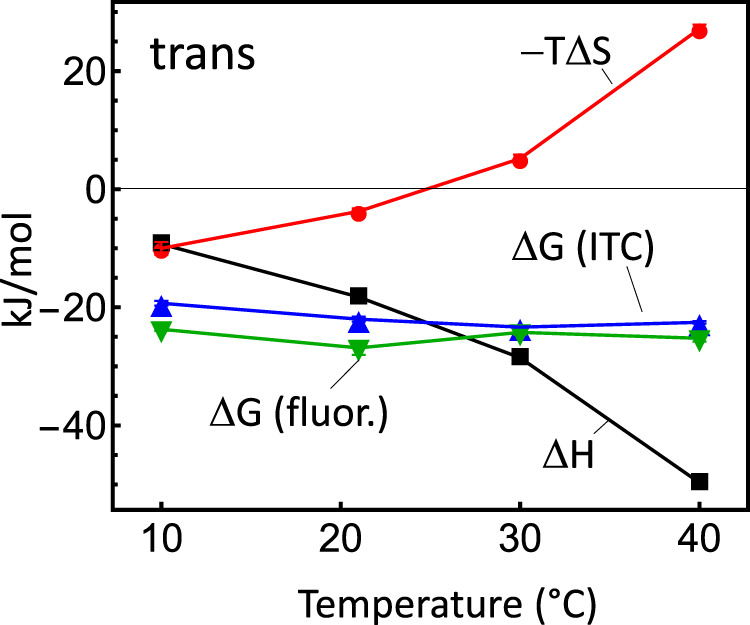
Temperature dependence of *ΔG*, *ΔH* and *−TΔS.* The binding free energy Δ*G* of the *trans*-state extracted from ITC (blue), along with the binding enthalpy Δ*H* (gray) and entropic contribution −*T*Δ*S* (red). The binding free energies deduced from the fluorescence depolarization experiments (green) are shown for comparison as well.

Figure 3 also compares the binding free energy determined from ITC (in blue) with that determined with the help of fluorescence depolarization (in green), revealing a good agreement. Unfortunately, we cannot investigate the *cis*-state with ITC, as that would require having the photoswitchable protein as the titrant in the syringe at mM concentrations, constantly illuminated during the experiment. Nevertheless, the good agreement of the *trans*-data gives us confidence that the *cis*-data of Fig. 2a and Table 1 are qualitatively correct as well.

## Consequences for the conclusions

The CD data in Fig. 2a of ref. ^1^, as well as the kinetic data in Figs. 2c, 3b of ref. ^1^ are not affected by the measurement error, since the protein and peptide concentrations, and hence the effect of reabsorption, are kept constant in either case. On the other hand, Fig. 2b of ref. ^1^ is wrong and has to be replaced by the new Fig. 2a presented here. There is no swap of binding affinity between *cis* and *trans*-state as a function of temperature, rather, the temperature dependence is weak (Fig. 3, blue and green). The *cis*-state has an about 8 ± 2 times larger binding affinity at all temperatures, which is still a sizeable effect, e.g., is an effect larger than that upon phosphorylation of the *α*3-helix.^2^ The Van-t’Hoff plot in Fig. 3a of ref. ^1^ is wrong and has to be replaced by Fig. 3, presented here.

Figure 4 replaces Fig. 4c of ref. ^1^. However, since we cannot disentangle enthalpic vs entropic contributions of the *cis*-state, Fig. 4 plots free energy profiles for the thermal *cis*-to-*trans* isomerization, rather than the enthalpy profiles in ref. ^1^. Since the binding energy is larger in the *cis*-state, the free energy driving force for isomerization is smaller in the ligand-bound state *PL*, yet the kinetics in this state is faster (see Figs. 2c, 3b of ref. ^1^). In the language of a Φ-value analysis, which we used in ref. ^1^, that situation results in a negative Φ-value. The other prominent example in this regard is the “inverted regime” of electron transfer. Both situations are relatively rare, but not unheard of^7–9^. It typically implies that the reaction does not proceed in a straightforward manner along a particular reaction coordinate. For example, in the inverted regime of electron transfer, the reaction first moves “backward” along a collective solvation coordinate until it reaches the transition state, from where it then proceeds forward toward the product state. In the concrete case here, it implies that the isomerization coordinate of the azobenzene moiety is orthogonal to that of ligand binding. Nonetheless, the Arrhenius plot in Fig. 3b of ref. ^1^ reveals that for temperatures <40 °C, the thermally driven *cis*−*trans* isomerization is faster with the ligand bound, hence, the reaction barrier against isomerization is lower in terms of free energy, according to an Eyring equation, $k={k}_{B}T/h\exp (-\Delta {G}^{\#}/{k}_{B}T)$. While we no longer attempt to quantify the size of the force that gives rise to this effect, the results show that such an allosteric force exists.

**Fig. 4:**
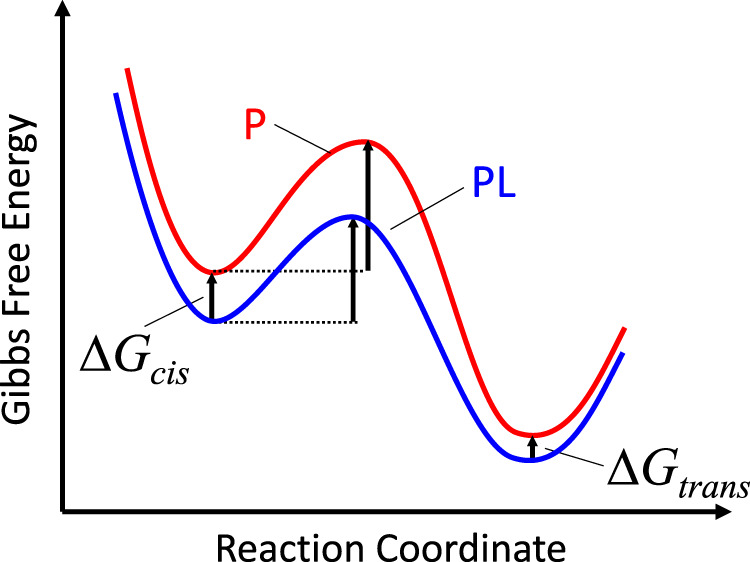
Free energy landscape of the thermal *cis*-to-*trans* isomerization of the free protein *P* (red) or the ligand-bound protein *PL* (blue).

Most importantly, the conclusions on bi-directionality of allosteric control remains, i.e., ligand binding affects the “reactivity” of the azobenzene moiety, and vice versa, the configuration of the azobenzene moiety significantly affects the binding affinity of the ligand. It is thus the smallest truly allosteric protein system, that, for example, is accessible to full-atom molecular dynamics simulations due to its small size and thus promises new insights into the microscopic understanding of allosteric communication. In summary, while many of the numbers in ref. ^1^ are wrong, the essential conclusions of the paper, i.e., bi-directional allosteric control, including the ability to sense the allosteric force, remain correct.
