# Supplementary material for: Sensing the allosteric force
Source: Nat Commun. 2020 Nov 17;11:5841. doi: 10.1038/s41467-020-19689-7 (PMC7673989; doi:10.1038/s41467-020-19689-7)
Supplement: Supplementary file 1 — Supplementary Information [file 41467_2020_19689_MOESM1_ESM.pdf]

Supplementary Information: Sensing the Allosteric Force

Olga Bozovic, Brankica Jankovic, Peter Hamm

Department of Chemistry, University of Zurich, Zurich, Switzerland

*peter.hamm@chem.uzh.ch

Gene variant	Sequence
wild type PDZ3	CATATGCATC ACCACCACCA CCACGGTAAC CTGTACTTCC AGGGTCTGGG CGAGGAAGAT ATCCCGCGTG AGCCGCGTCG TATCGTGATT CACCGTGGCA GCACCGGTCT GGGCTTTAAC ATCGTTGGTG GCGAGAACGG TGAAGGCATC TTCATTAGCT TTATTCTGGC GGGTGGCCCG GCGGACCTGA GCGGCGAGCT GCGTAAGGGC GATCAGATCC TGAGCGTGAA CGGTGTTGAC CTGCGTAACG CGAGCCACGA ACAAGCGGCG ATTGCGCTGA AAAACGCGGG CCAGACCGTG ACCATCATTG CGCAATACAA GCCGGAGGAA TATAGCCGTT TCGAAGCGAA ATAGCTCGAG
photoswitchable PDZ3	CATATGCATC ACCACCACCA CCACGGTAAC CTGTACTTCC AGGGTCTGGG CGAGGAAGAT ATCCCGCGTG AGCCGCGTCG TATCGTGATT CACCGTGGCA GCACCGGTCT GGGCTTTAAC ATCGTTGGTG GCGAGAACGG TGAAGGCATC TTCATTAGCT TTATTCTGGC GGGTGGCCCG GCGGACCTGA GCGGCGAGCT GCGTAAGGGC GATCAGATCC TGAGCGTGAA CGGTGTTGAC CTGCGTAACG CGAGCCACGA ACAAGCGGCG ATTGCGCTGA AAAACGCGGG CCAGACCGTG ACCATCATTG CGCAATACAA GCCGTGCGAG TATAGCCGTT TCGAATGCAA ATAGCTCGAG

TABLE S1. Sequences of genes cloned into pET-30a(+) plazmids that were used in this study.

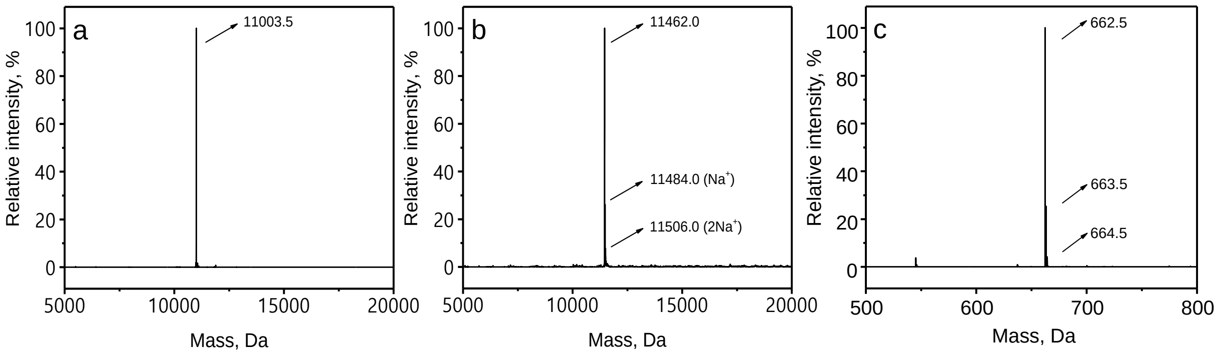

FIG. S1. Mass spectra for the wild type protein (a), photoswitchable protein (b) and peptide (c). Source data are provided as a Source Data file.

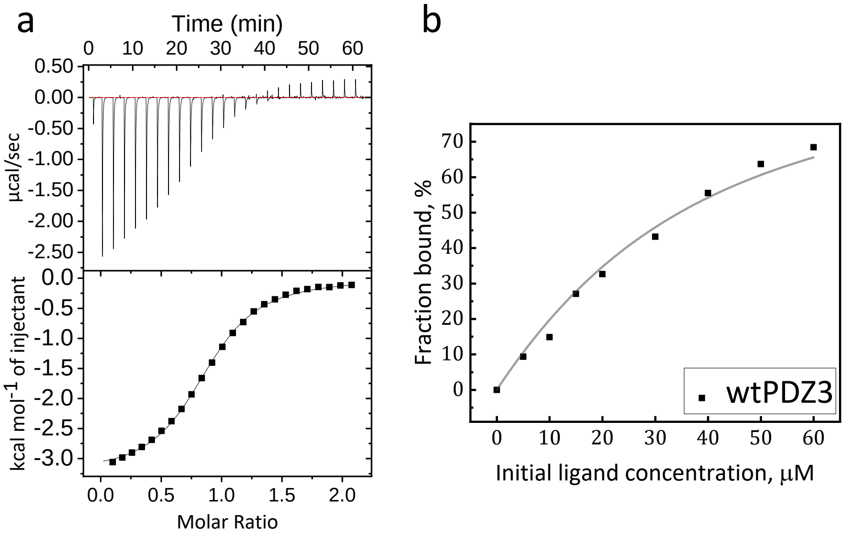

FIG. S2. (a) ITC thermogram for the wild type protein and the KETWV peptide, compared to (b) that determined from intrinsic fluorescence quenching. Source data are provided as a Source Data file.

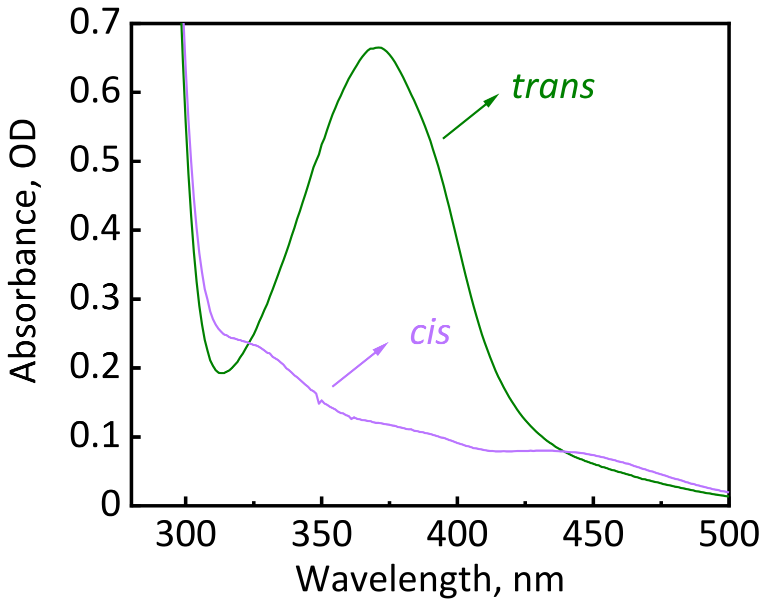

FIG. S3. UV/Vis spectra for the photoswitchable protein in *trans* (green) and *cis* state (purple). Source data are provided as a Source Data file.

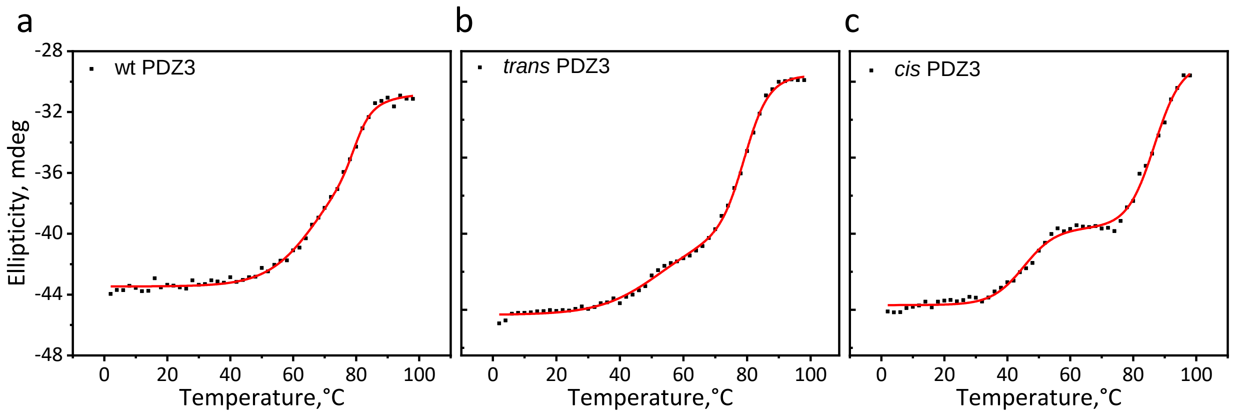

FIG. S4. Melting curve deduced from the CD at 220 nm for (a) the wild type, as well as (b) the *trans* and (c) the *cis* photoswitchable protein. Source data are provided as a Source Data file.

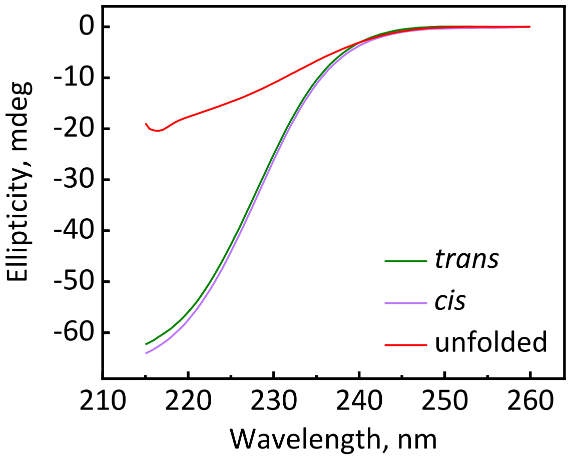

FIG. S5. CD spectra for the photoswitchable protein in *cis* (purple), *trans* (green) and unfolded protein (red). Source data are provided as a Source Data file.

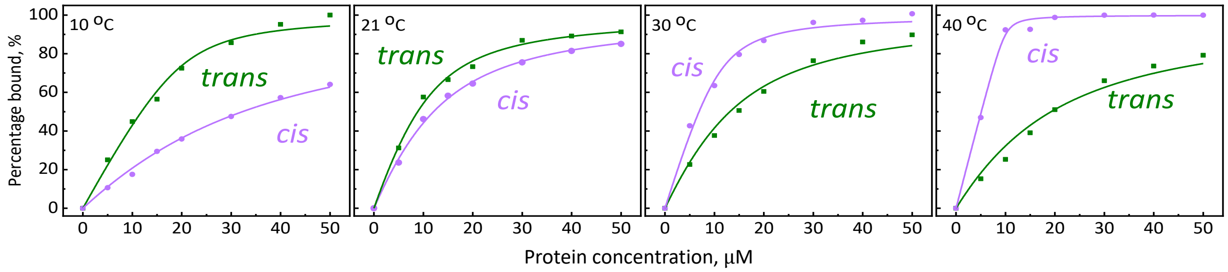

FIG. S6. Binding affinity determination using intrinsic fluorescence quenching at different temperatures for the *trans* (green) and *cis* (purple) photoswitchable protein. Source data are provided as a Source Data file.

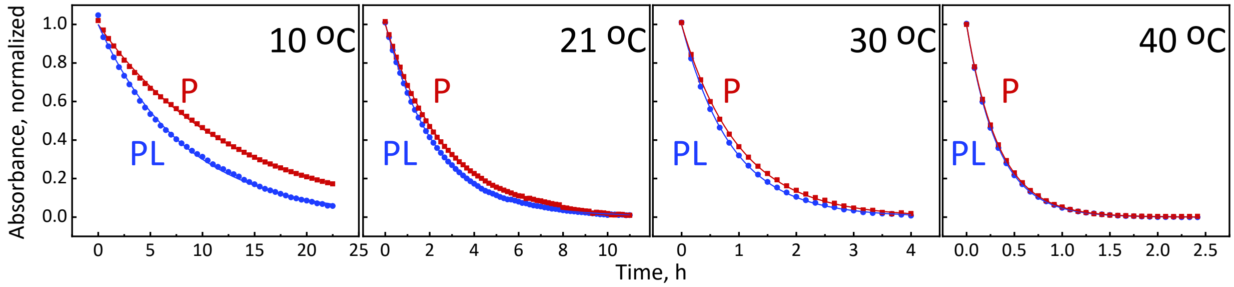

FIG. S7. UV/Vis spectra for the photoswitchable protein protein alone (red) and with ligand bound (blue). Source data are provided as a Source Data file.
